# Glycemic Control Improvement in Italian Children and Adolescents With Type 1 Diabetes Followed Through Telemedicine During Lockdown Due to the COVID-19 Pandemic

**DOI:** 10.3389/fendo.2020.595735

**Published:** 2020-12-07

**Authors:** Barbara Predieri, Francesco Leo, Francesco Candia, Laura Lucaccioni, Simona F. Madeo, Marisa Pugliese, Valentina Vivaccia, Patrizia Bruzzi, Lorenzo Iughetti

**Affiliations:** ^1^ Pediatric Unit, Department of Medical and Surgical Sciences of the Mother, Children and Adults, University of Modena and Reggio Emilia, Modena, Italy; ^2^ Post-Graduate School of Pediatrics, Department of Medical and Surgical Sciences for Mothers, Children and Adults, University of Modena and Reggio Emilia, Modena, Italy; ^3^ Pediatric Unit, Department of Pediatrics, Azienda Ospedaliero-Universitaria Policlinic, Modena, Italy; ^4^ Department of Metabolic Diseases and Clinical Nutrition, Azienda Ospedaliero-Universitaria Policlinic, Modena, Italy

**Keywords:** COVID-19, telemedicine—utilization, continuous glucose monitoring system, glycemic control, type 1 diabetes, children and adolescents

## Abstract

**Background/Objective:**

To minimize the wide spread of coronavirus disease (COVID-19) pandemic, Italy was placed in an almost complete lockdown state that forced people to “stay at home”. Aim of this study was to evaluate the effects of lockdown on glycemic control in children and adolescents with type 1 diabetes (T1D) followed through telemedicine.

**Subjects/Methods:**

This observational study involved patients with T1D using the real-time continuous glucose monitoring (CGM) Dexcom G6^®^. Ambulatory glucose profile data from the 3-months before schools closure (November 26, 2019–February 23, 2020; T0) and from the 3-months of consecutive lockdown (February 24–May 18, 2020; T1) were compared.

**Results:**

Sixty-two children and adolescents (11.1 ± 4.37 years, 50% males) with T1D (median time disease 3.67 years) were enrolled in the study. Insulin total daily dose was unchanged, while time spent on physical activities was decreased (p<0.0001). Despite the lack of statistical significance, median value of the glucose management indicator decreased from 7.4% to 7.25%. Glucose standard deviation (p<0.0001) and coefficient of variation (p=0.001) improved across the study. Median time in range increased from 60.5% to 63.5% (p=0.008), time above range decreased from 37.3% to 34.1% (p=0.048), and time below range decreased from 1.85% to 1.45% (p=0.001).

**Conclusions:**

Overall, in our children and adolescents with T1D glycemic control improved during lockdown. Despite patients were confined to their homes and limited to exercise, our data suggest that the use of real-time CGM, the continuous parental management, and the telemedicine can display beneficial effects on T1D care.

## Introduction

On January 9, 2019, the Chinese Center for Disease Control and Prevention reported the new strain of coronavirus, severe acute respiratory syndrome coronavirus-2 (SARS-CoV-2), as the causative agent of the coronavirus disease-2019 (COVID-19) that from China spread worldwide in few months (1). The World Health Organization (WHO) on March 11, 2020 declared COVID-19 a pandemic health emergency (2). At time of this paper submission, 21.516.760 cases of COVID-19 have been confirmed all over the world, including 766.663 deaths, as reported by the WHO (3). Italy was the first EU country to be affected by the COVID-19 outbreak and the first autochthonous case was confirmed on February 21, 2020 in Codogno (Lodi, Lombardia). To date, 253.915 total cases of COVID-19 with 35.396 (13.9%) deaths have been confirmed in Italy (4) and in our Region (Emilia-Romagna), one of the most affected by COVID-19, the latest data update reported 29.575 cases, including 4.286 deaths. One thousand and nine (3.4%) of confirmed cases were in young people (≤19 years old) and among these no death was recorded (5).

In order to reduce the spread of COVID-19, the Italian Government firstly imposed the closure of all school levels and organized sport activities in 5 Regions in Northern Italy (including Emilia-Romagna) from February 23, 2020 (6). The almost full lockdown began on March 10, 2020 when movement restrictions, self-isolation, and social distancing were imposed all over the country (7). Due to the gradual reduction of new COVID-19 cases, from 18 May to 3 June, 2020 the lockdown was gradually stopped (8). These lockdown policies disrupted and overloaded the organization of our National Health System (NHS). Routine healthcare activities were deferred, so outpatient services were closed, including those for patients with chronic diseases. The widespread use of technology in type 1 diabetes (T1D) care allowed the diabetes team a remote monitoring of many patients through cloud platforms. Telemedicine was demonstrated to be an effective tool for diabetes care because it allows healthcare professionals to keep in touch with T1D patients (9, 10). According to our regional authorizations, during lockdown we changed our clinical practices and routine interactions with patients, switching the face-to-face encounters to scheduled video or phone visits, as other diabetes centers already reported (11, 12). Through the telemedicine we monitored and supported patients at home to avoid possible repercussions on disease management.

During the lockdown for COVID-19 outbreak, children and adolescents experienced difficulties to comply with the long period. The availability of technology allowed them to continue school learning and to ensure social networks by minimizing negative emotions related to the social isolation (13). However, due to the suspension of outside structured physical and leisure activities, housebound children and adolescents with T1D needed to change their disease care, so concerns about possible harmful effects on glycemic control were arisen (14). To the best of our knowledge, studies on how the change in daily activities and lifestyles during lockdown has affected glycemic control in subjects with T1D are few and have small samples or short observation times (15–19).

The aim of this study was to investigate the effects of daily routine changes, due to the COVID-19 lockdown, on glycemic control in a cohort of children and adolescents with T1D using the real-time continuous glucose monitoring (CGM). This system allowed us to download detailed data from the generated Ambulatory Glucose Profile (AGP) report. The primary outcome was to compare AGP data between the 3-months pre-lockdown and the 3-months of “stay at home” period. Secondary end points were changes of the insulin requirement and the time spent on physical activity during the two periods. The number of acute diabetes-related events was also recorded.

## Materials and Methods

### Patients and Study Design

This is a real-life, retrospective, and longitudinal observational study. Subjects were enrolled among children and adolescents with T1D followed at the Pediatric Diabetes Clinic of the University of Modena and Reggio Emilia that belongs to the public NHS and is the only provincial referral Center for the diabetes care of patients aged ≤18 years. We offer a multidisciplinary approach according to International guidelines (20, 21) which costs are totally borne by the NHS. All recruited patients were on insulin therapy following the standard medical protocol (22, 23), together with recommendations for self-monitoring of glucose (23–25), and healthy lifestyle (26, 27). Inclusion criteria were: age ≥1 year at recruitment, school attendance (from kindergarten to high school), T1D diagnosed (28) in our Clinic, regular 3-months clinical follow up attendance, regular self-monitoring of glucose with real-time CGM system Dexcom G6^®^ (Dexcom Inc., San Diego, California, US), sensor data shared with our Clinic to allow the digital remote monitoring, sensor use ≥70% over the most recent 14 days before both February 24 and May 18, and no transition from multiple daily injections (MDI) to continuous subcutaneous insulin infusion (CSII) or vice versa during study periods. Exclusion criteria were: other types of diabetes, T1D onset and diagnosis in another Center, other types of sensor, patients using Dexcom G6^®^ receiver instead of mobile phones, and documented chronic complications.

**Figure 1:**
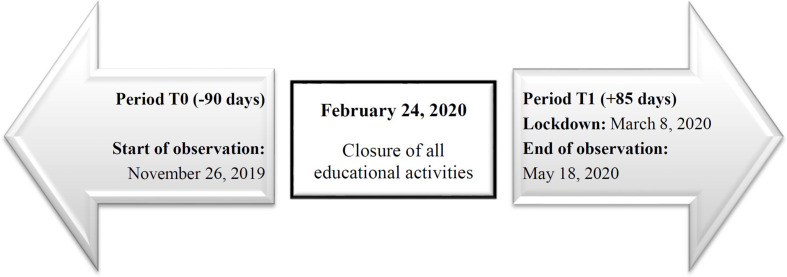
Timeline of study.

This study was started on February 24, 2020 when schools have been closed; all variables were collected in two different observation times (T) and were compared. We evaluated retrospectively the 3-months from November 26, 2019 to February 23, 2020 (T0) and longitudinally the 85 days of lockdown (T1: from February 24 to the end of the lockdown on May 18, 2020) (**Figure 1**). All data were recorded in the local pediatric diabetes registry (MyStar Connect data management software platform) and were anonymously recorded in a database using an alphanumeric and progressive identification code. During period T1 the healthcare staff provided diabetic, dietetic, psychological, and nursing assistance to all patients through telemedicine.

Our multidisciplinary team fulfill the educational training to all T1D patients and their caregivers at disease onset. The every 3-months follow-up includes the outpatient visit with the diabetologist and the nurse, while interviews with the dietician and the psychologist are carried out every 6 and 12 months, respectively, unless specific patients’ problems are identified. Considering the outstanding health crisis that we were experiencing due to the COVID-19 pandemic, all health professionals of the diabetes team had at least once telemedicine visit with all patients during period T1. Specifically, we stressed the importance to follow a balanced nutritional program (45%–55% of energy from carbohydrates, 25%–30% from fat, and 15%–20% from protein), to substitute high-glycemic index carbohydrate with low-glycemic index ones, to increase dietary fiber intakes, and to eat healthy snacks (27). Indications on how to reduce the daily energy and nutrient intakes were provided to patients who significantly reduced their time of weekly planned and structured physical activities during the lockdown, but growth, development, and good health characteristics were also considered in the dietetic approach for each child and teenager. Finally, patients that were educated in carbo-counting procedures, mainly CSII users, were helped to change insulin/carbohydrate ratio and insulin sensitivity factor, if necessary.

### Ethics Statement

This study was included in the existing observational study approved by the Ethics Committee of the University of Modena and Reggio Emilia (EC protocol number 330/16). Parental written informed consent, patient assent/consent, and online informed consent to be remotely connected to the diabetes clinic were obtained before starting the data collection.

### Anthropometry

Anthropometric measurements (height and weight) were collected retrospectively from data recorded during the last outpatient visit only in period T0. Measurements were performed according to the Anthropometric Standardization Reference Manual (29). Body mass index (BMI) was calculated from weight and height (kg/m^2^) and standardized to standard deviation (SD) score (BMI z-score) according to age and gender using appropriate Italian (30) and WHO growth charts (31). Pubertal status was assessed using the Tanner maturity scale (32).

### Glycemic Control

Patients’ sensor data were shared with our clinic and were downloaded from the commercially available web-based cloud platform (Dexcom Clarity^®^). We extracted data on glycemic control from the generated AGP report including: time below range (TBR; TBR^<70^ <70 mg/dl; TBR^<54^ <54 mg/dl), time in range (TIR^70-180^ 70–180 mg/dl), time above range (TAR; TAR^>180^ >180 mg/dl; TAR^>250^ >250 mg/dl); coefficient of variation (%CV), glucose management indicator (GMI); average glucose with SD, and sensor usage. Data were interpreted according to the International Consensus (33, 34).

Data on the time of weekly planned and structured physical activities (exercise, including the regularly one performed at home, i.e. jogging/running, bicycling/treadmill, aerobic play, dance, gymnastics, etc.) were also collected during outpatient visit at T0 and during the telemedicine visit at T1.

### Insulin Therapy

Insulin delivery method was categorized as MDI (≥4 injection time-points/day) or CSII (22, 23). Data on insulin doses were reported as units (IU) per kg per day. Insulin CSII data were uploaded online to the website Diasend^®^ platform, while insulin doses in MDI patients were obtained from the reading of the glycemic diary filled in by patients/parents or were extracted from the online Dexcom Clarity^®^ platforms.

During telemedicine visit at T1, we underlined the importance to deliver insulin dose at the right time before meals to improve post-prandial glycemia (22, 27).

### Acute Diabetes-Related Complications

Severe hypoglycemic and diabetic ketoacidosis (DKA) events during both the T0 and the T1 period were also collected. Severe hypoglycemia was defined as an event associated with severe cognitive impairment requiring assistance to administer glucagon or take other corrective actions (35). DKA was defined according to clinical signs associated to biochemical criteria (36).

### Statistical Analysis

Data were checked for normal distribution using the Kolmogorov-Smirnov test. Continuous data are reported as mean ± SD, and median, while categorical as absolute frequencies and percent values. The Wilcoxon matched pairs test was used for longitudinal comparison of variables recorded before and after the lockdown, the Mann-Whitney’s *U*-test for between-group analysis, and the Pearson χ^2^ for categorical variables when appropriate. Spearman correlation was used to identify the relationship between changes in physical activity with changes in glycemic control metrics during lockdown frame.

Statistical analysis was performed using the STATISTICA™ software (StatSoft Inc., Tulsa, OK, USA). For each test, statistical significance was considered for p <0.05.

## Results

Sixty-two children and adolescents with T1D (11.1 ± 4.37 years) were enrolled in the study. Half of the participants were males, pre-pubertal status was recorded in 54.8%, and the T1D duration was 4.89 ± 4.23 years. The median height was 0.14 SDS and the median BMI z-score was 0.05 SDS. Twenty-nine out of 62 subjects (46.8%) were CSII users (**Table 1**). Specifically, 13 out of 29 were using the t:slim X2^™^ pump with basal-IQ predictive low-glucose-suspend system (Tandem Diabetes Care, San Diego, CA, US). The other pumps were non-automated insulin delivery systems including 10 Accu-Chek Insight^®^ pumps (Roche Diagnostics GmbH, Mannheim, Germany) and 6 Omnipod^®^ patch pumps (Insulet Corporation, Acton, MA, US). Aspart insulin was used in all durable devices, while lispro was used in patch pumps. All the other patients were on MDI with a daily dose of long-acting analogue (glargine or degludec), regular insulin twice a day before breakfast and lunch, and a daily short-acting analogue (lispro) before dinner. No patient was hospitalized because of COVID-19 nor was quarantined for close contact with SARS-CoV-2 infected people. Moreover, severe hypoglycemic and DKA events were never reported during the study.

**Table 1:** Clinic, therapy, and glycemic control parameters in children and adolescents with T1D.

Variable/Observation period	T0	T1	p
Gender (Males/Females) (%)	31/31 (50/50)	31/31 (50/50)	–
Age (years)	11.1 ± 4.37 (10.9)	11.4 ± 4.37 (11.2)	<0.0001
T1D time disease (years)	4.89 ± 4.23 (3.67)	5.19 ± 4.22 (3.97)	<0.0001
Puberty (NO/YES) (%)	34/28 (54.8/45.2)	34/28 (54.8/45.2)	–
Insulin delivery method (MDI/CSII) (%)	33/29 (53.2/46.8)	33/29 (53.2/46.8)	–
Meal-Time insulin (IU/kg/day)	0.40 ± 0.14 (0.38)	0.40 ± 0.13 (0.38)	0.546
Basal insulin (IU/kg/day)	0.32 ± 0.13 (0.33)	0.34 ± 0.13 (0.34)	0.027
TDD insulin (IU/kg/day)	0.72 ± 0.22 (0.73)	0.74 ± 0.19 (0.77)	0.186
Physical Activity (h/week)	3.27 ± 2.82 (2.00)	0.24 ± 0.59 (0.00)	<0.0001
GMI (%); (mmol/mol)	7.45 ± 0.74 (7.40); 57.9 ± 8.13 (57.4)	7.35 ± 0.72 (7.25); 56.9 ± 7.89 (55.7)	0.069; 0.071
GMI ≤7.0% (YES/NO) (%)	22/40 (35.5/64.5)	21/41 (33.9/66.1)	χ^2^ = 34.9; p <0.0001
Average glucose (mg/dl)	167.4 ± 21.6 (166.0)	164.6 ± 21.1 (162.0)	0.058
SD glucose (mg/dl)	60.8 ± 11.8 (61.5)	57.6 ± 10.8 (58.0)	<0.0001
%CV (%)	36.3 ± 5.31 (36.1)	34.9 ± 4.90 (34.0)	0.001
TBR^<70^ (%); TBR^<54^ (%)	2.63 ± 2.37 (1.85); 0.50 ± 0.63 (0.30)	2.13 ± 2.41 (1.45); 0.34 ± 0.53 (0.20)	0.001; 0.002
TIR^70-180^ (%)	60.0 ± 13.1 (60.5)	62.1 ± 13.7 (63.5)	0.008
TAR^>180^ (%); TAR^>250^ (%)	37.8 ± 13.9 (37.3); 11.4 ± 7.77 (10.0)	35.7 ± 14.4 (34.1); 9.74 ± 7.00 (8.95)	0.048; <0.001
Sensor usage (%)	92.0 ± 11.3 (96.5)	91.8 ± 12.1 (96.5)	0.755

Continuous data are reported as mean ± SD (median) ; categorical as absolute frequencies (percent values).

CSII, continuous subcutaneous insulin infusion; CV, coefficient of variation; GMI, glucose management indicator; MDI, multiple daily injections; SD, standard deviation; T1D, type 1 diabetes; TAR, time above range; TBR, time below range; TDD, total daily dose; TIR, time in range.

All patients and/or their caregivers were managed using telemedicine during lockdown. Video calls were performed 47.0 ± 21.6 days after the beginning of lockdown (median 48.5 days); 21 out of 62 patients had the scheduled video call by the end of March and they were contacted by the diabetologist with a second call in early May.

**Figure 2:**
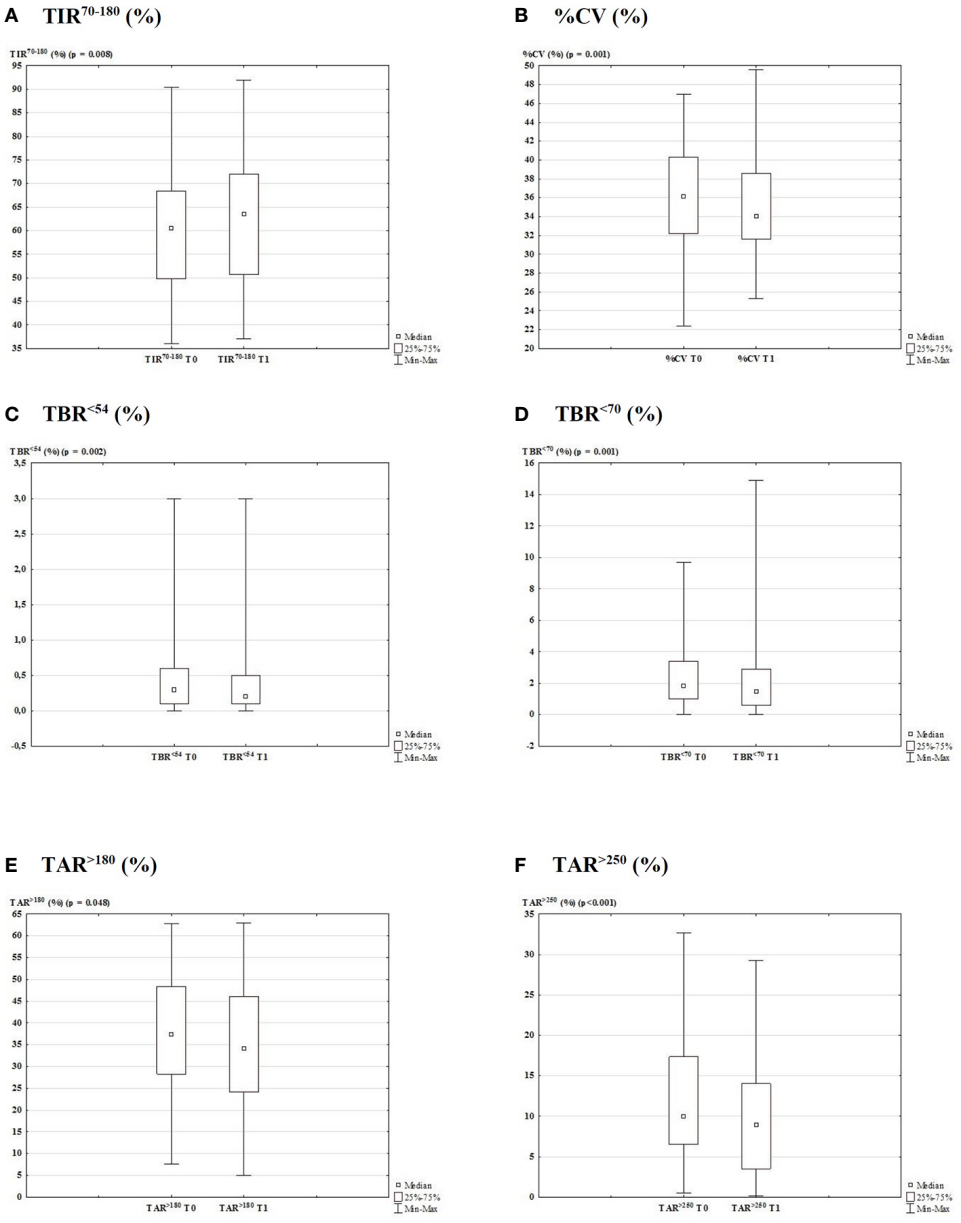
Glycemic control data generated from the AGP report 3-months before and after lockdown due to COVID-19 outbreak (T0 vs. T1). Box plot with the percentage of TIR^70-180^ (**A**; p = 0.008), CV (**B**; p = 0.001), TBR^<54^ (**C**; p = 0.002), TBR^<70^ (**D**; p = 0.001), TAR^>180^ (**E**; p = 0.048), and TAR^>250^ (**F**; p < 0.001).

Detailed glycemic control and therapeutic data are reported in **Table 1** and **Figure 2** depicts the metrics from the generated AGP report. We found that the mean values of TIR^70-180^ significantly increased from T0 to T1 (60.0 ± 13.1 vs. 62.1 ± 13.7%, respectively; p = 0.008; **Figure 2A**). It was unchanged in 9.7% (n = 6) of patients, while an improvement >1% was found in 64.5% (n = 40) and a deterioration >1% in 25.8% (n = 16). The %CV was also improved from T0 to T1 (36.3 ± 5.31 vs. 34.9 ± 4.90%, respectively; p = 0.001; **Figure 2B**), resulting unchanged in 17.7% (n = 11) of patients, improved by >1% in 59.7% (n = 37), and deteriorated by >1% in 22.6% (n = 14). Across the study, from T0 to T1, we found a significant decrease in TBR^<54^ (0.50 ± 0.63 vs. 0.34 ± 0.53%, respectively; p = 0.002; **Figure 2C**), TBR^<70^ (2.63 ± 2.37 vs. 2.13 ± 2.41%, respectively; p = 0.001; **Figure 2D**), TAR^>180^ (37.8 ± 13.9 vs. 35.7 ± 14.4%, respectively; p = 0.048; **Figure 2E**), and TAR^>250^ (11.4 ± 7.77 vs. 9.74 ± 7.00%, respectively; p <0.001; **Figure 2F**) values. The average glucose was unchanged, while the median SD of sensor readings decreased from 61.5 mg/dl to 58.0 mg/dl (p <0.0001).

Despite the lack of statistical significance, GMI median values slightly decreased from period T0 to T1 (7.40 vs. 7.25%, respectively; p = 0.069). We found that four out of 22 patients with GMI ≤7.0% at T0 were worsened at T1 and three out of 40 patients having GMI >7.0% at T0 improved in period T1 (χ^2^ = 34.9; p <0.0001). All patients stopped outside structured physical activities during T1 period, but they tried to maintain the same regular exercise they already did when were at home during T0. So, we found that time spent on exercise was significantly lower at T1 compared to T0 (0.24 ± 0.59 vs. 3.27 ± 2.82 h/week, respectively; p <0.0001), but the insulin total daily dose (TDD) did not significantly change longitudinally.

Sensor usage <70% was found in four out 62 patients at T0 and in five subjects during T1 period, so the CGM usage did not significantly change and the sensor was worn for a median time of 96.5% in both periods.

Lastly, we did not find significant correlation between the change of the time spent on physical activities (median Δ = -2.00 h/week) and changes of each glycemic control metric.

All data were also analyzed according to gender (**Table S1**), pubertal status (**Table S2**), and insulin delivery methods (**Table S3**).

No gender-specific significant differences were observed in glycemic control metrics during both periods’ study (**Table S1**).

Glycemic control data were comparable between pre-pubertal and pubertal patients at period T0, while at the end of the lockdown it was worse in pubertal patients than in pre-pubertal ones, as demonstrated by lower median values of TIR^70-180^ (53.9 vs. 65.7%, respectively; p = 0.025) and higher median values of TAR^>180^ (43.7 vs. 32.0%, respectively; p = 0.015), GMI (7.75 vs. 7.20%, respectively; p = 0.011), and average glucose (175.6 vs. 159.5 mg/dl, respectively; p = 0.013) (**Table S2**). Conversely, median values of TBR^<70^ (1.10 vs. 2.00%, respectively; p = 0.002) and %CV (33.2 vs. 35.1%, respectively, p = 0.047) were lower. Analyzing pre-pubertal and pubertal groups separately, in both groups we demonstrated that, from period T0 to period T1, the %CV, the SD of sensor readings, and the time spent on exercise were significantly decreased (p <0.05). An increase of TIR^70-180^ values (from 62.7 ± 12.3 to 65.6 ± 11.4%; p = 0.003) as well as a decrease of TAR^>180^ (from 34.1 ± 12.9 to 31.5 ± 12.1%, p = 0.024) were found only in the pre-pubertal group, while TBR^<70^ values were decreased only in the pubertal group (from 1.99 ± 2.18 to 1.24 ± 1.12%; p = 0.003). All the other AGP metrics did not significantly change (**Table S2**).

Lastly, glycemic metrics at the end of lockdown were not different between patients who were on the MDI and those who were on the CSII (**Table S3**). The intra-group analysis allowed us to identify that in both groups the time spent on exercise and the SD of sensor readings were significantly decreased (p <0.05) from period T0 to period T1. The %CV (from 37.4 ± 4.88 to 35.0 ± 3.40%; p <0.001) and the TBR^<70^ (from 3.11 ± 2.5 to 1.98 ± 1.48%; p <0.0001) values were decreased only in CSII group, while the TAR^>250^ decreased only in MDI group (from 11.5 ± 8.36 to 9.31 ± 6.78%; p <0.001).

## Discussion

To the best of our knowledge, this real-life observational study is the first one evaluating the impact of the entire lockdown period due to the COVID-19 pandemic on glycemic control in children and adolescents with T1D which were using the real-time CGM system Dexcom G6^®^ and were followed through telemedicine. We found a significant improvement of the glycemic control, as suggested by the increase of TIR^70-180^ values and the reduction of both TBR and TAR values. The glucose variability decreased and remained below the established target of 36% (34), while the median GMI value we reported at the end of the lockdown frame (7.25%) was comparable to that recorded in the 3-months before (7.40%). These findings are amazing considering that children and adolescents with T1D did not change insulin TDD and had significantly lesser opportunities to perform physical activities. Despite patients had no access to outpatient visits because of housebound, they interacted with the multidisciplinary diabetes team through telemedicine and the real-time CGM system together with the remote control access allowed us to report a large number data on glycemic control.

Measures issued by governments were necessary to avoid COVID-19 spread, but there were concerns that the prolonged school closure, the housebound, the limited possibility to exercise, and the psychologic stress imposed by social distancing could have had negative consequences on glycemic control in patients with T1D (37, 38). The main goal of the T1D therapy is to reach a good glycemic control (25, 39), in order to prevent short- and long-term complications (40). The beneficial effect of physical activity on glycosylated hemoglobin (HbA1c) has been extensively investigated and a meta-analysis showed an overall effect on HbA1c of -0.85% (14). Data we found in our children and adolescents disagree with that obtained in adults with T1D in which it was reported a decrease in exercise and a worst in glucose values (15, 41). Again, glycemic control in adolescents with T1D using hybrid closed loop system did not worsen during the restrictions due to COVID-19 pandemic and further improved in those who continued physical activity during the first 2-week of quarantine, confirming the importance of maintaining a regular physical activity also at home (19).

An encouraging aspect that emerges from our patients during the lockdown frame is that they were able to prevent the worsening of glycemic control without the necessity to increase insulin TDD regardless of gender, pubertal status, and insulin delivery method although they were much more sedentary. However, during telemedicine visit, we stressed the importance to keep a balanced nutritional program according to the new time spent on exercise and we helped patients to change their insulin/carbohydrate ratio and insulin sensitivity factor. Our observations are reassuring and suggest that a slowing down of routine activities can have favorable effects on the glycemic control. We can hypothesize that this improvement, mainly in young children, could result from the opportunity of a more regular lifestyle, including scheduled mealtimes without higher food intake and more frequent snacking, and more time to concentrate on T1D care by parents who were forced to “stay home” and by children who were stopped to attend the school. Moreover, the well-known difficulties to appropriately modulate carbohydrate intakes and insulin doses in relation to exercise must to be taken into consideration. Again, elevation of blood glucose level predicted worse outcomes in hospitalized patients with COVID-19 (42), so the knowledge that diabetes worsens the outcomes of COVID-19 (43–45) may have improved patients’ awareness and compliance to diabetes management.

We showed a better glycemic control in pre-pubertal patients, compared to those who were in pubertal development status, as well as the significant increase of the TIR^70-180^ and the significant decrease of the TAR from T0 to T1 were found only in the pre-pubertal group. Moreover, in our patients the median %CV decreased from 36% to 34% and this improvement was found in both subgroups. Young people with T1D spend many hours at school, so trained school-staff is important to provide a safe environment and it plays a key role in reducing glycemic variability, mainly in younger children (46, 47). Our data during the pre-lockdown school period demonstrate a relatively good glycemic control (median GMI 7.4%, TIR 60.5%, and %CV 36.1%) and allow us to confirm that the real-time CGM systems associated with the remote control access improved the treatment and management of T1D during school hours. In Italy, the widespread use of CGM and flash glucose monitoring among people with T1D allowed these patients to be remotely connected to the clinic through the cloud, but the use of these technologies in children and adolescents have also bridged the gap between school and family care capacities. Recently, it was demonstrated that parents of kindergarten and school children reported the usefulness and the effectiveness of remote monitoring and CGM to control glucose excursions (48). The COVID-19 pandemic has forced children and parents to spend many hours together at home, bringing back diabetes care to parents. Our observational real-life study confirms the positive effect of parental care in T1D young children, regardless of the use of new technologies as previously demonstrated (18, 49). We can speculate that similar results were not achieved in adolescents because the management of the disease in these patients remained in their hands even during confinement. However, in our adolescents the lockdown frame had no pejorative effect on glycemic control. Adolescence is a well-known, high-risk time period for all youths who experience rebellion and lawlessness. Despite the continuous advancements in diabetes care, the achievement of a good glycemic control represents a complex task for patients with T1D, especially for adolescents (50, 51). We can speculate that children were mainly affected by the quarantine as they are still in need for reassurance and parental care, and appear uncertain in the management of the disease because of poor autonomy, while adolescents were not affected by the quarantine period in their approach to the disease.

The real-world data highlighted that patients using CSII have a better short- and long‐term glycemic control relative to matched MDI ones (52). However, we found that glycemic metrics at the end of the lockdown were not different between patients who were on MDI and those who were on CSII. Despite the TIR^70-180^ was unchanged from period T0 to period T1 in both groups, TBR and %CV values were significantly decreased in CSII users, while the TAR^>250^ improved in MDI group. The decrease in TBR we found in CSII group can be explained by the fact that 16 out of 29 children were using non-automated insulin delivery systems and by the greater parental T1D management in these patients. Our 3-months effects of lockdown on glycemic control are similar to those evaluated in the study from Greece including children with T1D wearing CSII equipped with CGM. In this study data of 3-weeks before and after lockdown were compared and no significant difference in insulin TDD and TIR values was demonstrated. The %CV values significantly decreased, but they were reported to be above 36% (53). During the first 2-weeks lockdown period, as compared to the 2-weeks previous, higher TIR values and lower TAR values were demonstrated in Italian pre-school and school T1D children using CGM and semi-automated insulin delivery systems, while TBR was unchanged. However, in this study results were probably due to a significant increase in insulin boluses (18).

Despite difficulties, both video and phone calls allowed us to conduct virtual visits with patients and their caregivers and in our experience, telemedicine was used safely and effectively, as previously reported (9, 10). Evidences supporting the use of telemedicine for glycemic control in patients with T1D are still lacking (54), while its emerging importance was reported for the management of other chronic disorders. In patients with type 2 diabetes in a rural community, telemedicine was found to be a useful tool to share educational and treatment approaches with the diabetes team, also improving HbA1c values (55). Telemedicine tools were also demonstrated to be adaptable for direct consultation in other healthcare environments (56, 57) and in access-poor populations (58). Through telemedicine, we maintained social distancing, minimized the risk of SARS-CoV-2 transmission, and suggested to patients the strategies to pursue the better glycemic control. We reinforced the importance of non-omission of insulin, healthy eating, continuing physical activity at home, and remaining hydrate to prevent acute complications and to avoid visit to the Emergency Department. None of our patients were hospitalized because of COVID-19 nor were quarantined for close contact with SARS-CoV-2 infected people. To date, few data on European pediatric COVID-19 have been published, but specific management strategies were recommended (59). Children and adolescents seem to be less often affected and to experience a milder form of illness compared to adults (60–63). However, uncertainties on treatment options in the pediatric age are still present. COVID-19 recommendations for children with T1D were also reported (64), but children with T1D have not shown different disease pattern or susceptibility to SARS-CoV-2 infection compared to healthy peers (65). As published in a recent survey, the majority of worldwide diabetes centers did not have COVID-19 in children with T1D and few cases with mild or moderate disease course were reported (12).

Finally, in our study glycemic metrics were improved without any outpatient visits and severe hypoglycemic and DKA events were never reported during the two periods. They were probably prevented by the shared glucose data through Dexcom Clarity^®^ and the use of telemedicine.

The main strength of our study is the assessment of outcomes during the entire lockdown period. Moreover, we decided strict inclusion criteria and recruited in the study only patients using one specific real-time CGM to avoid the discrepancies in CGM-derived GMI values between different sensor types (66) and the false CGM reading due to acetaminophen interference (67), if administered for any reason.

The study has some limitations: first, it is a single-center study; secondly, diet characteristics were not fully analyzed. We acknowledge that detailed information to interpret the drivers of glucose control during lockdown, such as consumption of snacks, were not available and should be investigated in future studies. Moreover, our data refer to children and adolescents with T1D having a relatively good glycemic control and showing frequent sensor use prior the inclusion in the study, so results may not be generalizable to patients with poorer control or not using real-time CGM. On the other hand, the COVID-19 pandemic has highlighted disparities between those who do and do not have access to technology (68).

In conclusion, despite the limitations of lockdown due to the COVID-19 pandemic and the significant decrease of time spent on physical activity, we report an improvement of glycemic control and no increase in acute complications events in children and adolescents with T1D using real-time CGM during the 3-months lockdown. These results seem to be very encouraging and highlight the importance of a more stable rhythm of life and a more constant parental diabetes care in young children regardless from gender, pubertal status, and method of insulin administration. Finally, our study emphasizes the usefulness of telemedicine in situations of forced isolation. We live in a world where the web access is now a necessity and the advancement of continuously connected devices allows the easy flow of data from patient to physician, for those using such devices, during a health crisis period. In our study we used instruments that are already adopted in many other Diabetes Centers. Therefore, we believe that our results offer a reproducible model useful for the future clinical practice during other pandemic events and for further researches, not only in the diabetic field but probably also in other chronic diseases.

## Data Availability Statement

The data analyzed in this study is subject to the following licenses/restrictions: Datasets consist of data routinely recorded in clinical practice and anonymously recorded. Requests to access these datasets should be directed to Barbara Predieri, barbara.predieri@unimore.it.

## Ethics Statement

The studies involving human participants were reviewed and approved by Ethics Committee of the University of Modena and Reggio Emilia. Written informed consent to participate in this study was provided by the participants’ legal guardian/next of kin.

## Author Contributions

BP acts as guarantor for the contents of this article. BP and LI designed and coordinated the research study. BP and FL wrote the paper and performed the data analyses. BP, LL, and PB contributed to the interpretation of data and performed a critical revision of content. SM, MP, and VV contributed to the literature search. FL and FC collected data. All authors contributed to the article and approved the submitted version.

## Conflict of Interest

The authors declare that the research was conducted in the absence of any commercial or financial relationships that could be construed as a potential conflict of interest.

## Abbreviations

AGP, ambulatory glucose profile; BMI, body mass index; CGM, continuous glucose monitoring; COVID-19, coronavirus disease; CSII, continuous subcutaneous insulin infusion; CV, coefficient of variation; DKA, diabetic ketoacidosis; GMI, glucose management indicator; MDI, multiple daily injections; NHS, National Health System; SARS-CoV-2, severe acute respiratory syndrome coronavirus-2; SD, standard deviation; SDS, standard deviation score; T1D, type 1 diabetes; TAR, time above range (both >180 and >250 mg/dl); TAR^180^, time above range >180 mg/dl; TAR^250^, time above range >250 mg/dl; TBR, time below range (both <54 and <70 mg/dl); TBR^54^, time below range <54 mg/dl; TBR^70^, time below range <70 mg/dl; TDD, total daily dose; TIR^70-180^, time in range 70–180 mg/dl; WHO, World Health Organization.

## References

[1] Zhu N Zhang D Wang W Li X Yang B Song J A novel coronavirus from patients with pneumonia in China, 2019. N Engl J Med (2020) 382:727–33. 10.1056/NEJMoa2001017 PMC7092803 31978945

[2] World Health Organization (WHO) “WHO Director-General’s opening remarks at the media briefing on COVID-19 - 11 March 2020. Available at: https://www.who.int/dg/speeches/detail/who-director-general-s-opening-remarks-at-the-media-briefing-on-covid-19—11-march-2020 (Accessed August 3, 2020).

[3] World Health Organization (WHO) WHO coronavirus disease (COVID-19) Dashboard. Available at: https://covid19.who.int/ (Accessed August 17, 2020).

[4] Department of Civil Protection COVID-19 case update. Available at: http://opendatadpc.maps.arcgis.com/apps/opsdashboard/index.html#/b0c68bce2cce478eaac82fe38d4138b1 (Accessed August 17, 2020).

[5] Emilia-Romagna Region Coronavirus: measurements in Emilia-Romagna. Info-graphics. Available at: https://www.regione.emilia-romagna.it/coronavirus/infografiche (Accessed August 17, 2020).

[6] Decree Law of the Italian Prime Minister Urgent measures on the containment and management of the epidemiological emergency by COVID-19. Available at: https://www.gazzettaufficiale.it/eli/id/2020/02/23/20G00020/sg (Accessed August 3, 2020). February 23, 2020.

[7] Decree Law of the Italian Prime Minister Further measures for the containment and contrast of the spread of the Covid-19 virus throughout the entire national territory. Available at: https://www.gazzettaufficiale.it/eli/id/2020/03/09/20G00030/sg (Accessed August 3, 2020). March 9, 2020.

[8] Decree Law of the Italian Prime Minister Additional urgent measures to deal the COVID-19 epidemiological emergency. Available at: https://www.gazzettaufficiale.it/eli/id/2020/05/17/20A02717/sg (Accessed August 3, 2020). May 17, 2020.

[9] Giani E Laffel L Opportunities and challenges of telemedicine: observations from the wild west in pediatric type 1 diabetes. Diabetes Technol Ther (2016) 18:1–3. 10.1089/dia.2015.0360 26756102 PMC5248506

[10] McDonnell ME Telemedicine in complex diabetes management. Curr Diabetes Rep (2018) 18:42–50. 10.1007/s11892-018-1015-3 29797292

[11] Clary L Wang C Byrne ME Monaghan M COVID-19 pandemic-related practices and policies affecting the continuity of behavioral health care among children with diabetes. Transl Behav Med (2020) 10:819–26. 10.1093/tbm/ibaa072 PMC7529096 32710626

[12] Elbarbary NS Dos Santos TJ de Beaufort C Agwu JC Calliari LE Scaramuzza AE COVID-19 outbreak and pediatric diabetes: perceptions of health care professionals worldwide. Pediatr Diabetes (2020). 10.1111/pedi.13084 PMC7404589 32686287

[13] Goldschmidt K The COVID-19 pandemic: technology use to support the wellbeing of children. J Pediatr Nurs (2020) 53:88–90. 10.1016/j.pedn.2020.04.013 32317129 PMC7161478

[14] MacMillan F Kirk A Mutrie N Matthews L Robertson K Saunders DH A systematic review of physical activity and sedentary behavior intervention studies in youth with type 1 diabetes: study characteristics, intervention design, and efficacy. Pediatr Diabetes (2014) 15:175–89. 10.1111/pedi.12060 23895512

[15] Assaloni R Pellino VC Puci MV Ferraro OE Lovecchio N Girelli A Coronavirus disease (Covid-19): how does the exercise practice in active people with type 1 diabetes change? A preliminary survey. Diabetes Res Clin Pract (2020) 166:108297. 10.1016/j.diabres.2020.108297 32623042 PMC7332427

[16] Bonora BM Boscari F Avogaro A Bruttomesso D Fadini GP Glycaemic control among people with type 1 diabetes during lockdown for the SARS-CoV-2 outbreak in Italy. Diabetes Ther (2020) 11:1–11. 10.1007/s13300-020-00829-7 PMC7213551 32395187

[17] Murphy HR Managing diabetes in pregnancy before, during, and after COVID-19. Diabetes Technol Ther (2020) 22:454–61. 10.1089/dia.2020.0223 32396397

[18] Schiaffini R Barbetti F Rapini N Inzaghi E Deodati A Patera IP School and pre-school children with type 1 diabetes during covid-19 quarantine: the synergic effect of parental care and technology. Diabetes Res Clin Pract (2020) 166:108302. 10.1016/j.diabres.2020.108302 32623034 PMC7332425

[19] Tornese G Ceconi V Monasta L Carletti C Faleschini E Barbi E Glycemic control in type 1 diabetes mellitus during COVID-19 quarantine and the role of in-home physical activity. Diabetes Technol Ther (2020) 22:462–7. 10.1089/dia.2020.0169 32421355

[20] Young-Hyman D de Groot M Hill-Briggs F Gonzalez JS Hood K Peyrot M Psychosocial care for people with diabetes: a position statement of the American Diabetes Association. Diabetes Care (2016) 39:2126–40. 10.2337/dc16-2053 PMC5127231 27879358

[21] Delamater AM de Wit M McDarby V Malik JA Hilliard ME Northam E ISPAD Clinical Practice Consensus Guidelines 2018: psychological care of children and adolescents with type 1 diabetes. Pediatr Diabetes (2018) 19:237–49. 10.1111/pedi.12736 30058247

[22] Danne T Phillip M Buckingham BA Jarosz-Chobot P Saboo B Urakami T ISPAD Clinical Practice Consensus Guidelines 2018: insulin treatment in children and adolescents with diabetes. Pediatr Diabetes (2018) 19:115–35. 10.1111/pedi.12718 29999222

[23] Sherr JL Tauschmann M Battelino T de Bock M Forlenza G Roman R ISPAD Clinical Practice Consensus Guidelines 2018: Diabetes technologies. Pediatr Diabetes (2018) 19:302–25. 10.1111/pedi.12731 30039513

[24] Scaramuzza A Cherubini V Tumini S Bonfanti R Buono P Cardella F Diabetes Study Group of the Italian Society of Pediatric Endocrinology and Diabetology. Recommendations for self-monitoring in pediatric diabetes: a consensus statement by the ISPED. Acta Diabetol (2014) 51:173–84. 10.1007/s00592-013-0521-7 24162715

[25] DiMeglio LA Acerini CL Codner E Craig ME Hofer SE Pillay K ISPAD Clinical Practice Consensus Guidelines 2018: glycemic control targets and glucose monitoring for children, adolescents, and young adults with diabetes. Pediatr Diabetes (2018) 19:105–14. 10.1111/pedi.12737 30058221

[26] Adolfsson P Riddell MC Taplin CE Davis EA Fournier PA Annan F ISPAD Clinical Practice Consensus Guidelines 2018: exercise in children and adolescents with diabetes. Pediatr Diabetes (2018) 19:205–26. 10.1111/pedi.12755 30133095

[27] Smart CE Annan F Higgins LA Jelleryd E Lopez M Acerini CL ISPAD Clinical Practice Consensus Guidelines 2018: nutritional management in children and adolescents with diabetes. Pediatr Diabetes (2018) 19:136–54. 10.1111/pedi.12738 30062718

[28] American Diabetes Association: 2 Classification and diagnosis of diabetes: standards of medical care in diabetes - 2020. Diabetes Care (2020) 43:S14–31. 10.2337/dc20-S002 31862745

[29] Lohman TG Roche AF Martorell R eds. Anthropometric Standardization Reference Manual. 1st ed. Champaign, IL: Human Kinetics Books (1988).

[30] Cacciari E Milani S Balsamo A Spada E Bona G Cavallo L Italian cross-sectional growth charts for height, weight and BMI (2 to 20 yr). J Endocrinol Invest (2006) 29:581–93. 10.1007/BF03344156 16957405

[31] WHO Multicentre Growth Reference Study Group WHO Child Growth Standards based on length/height, weight and age. Acta Paediatr Suppl (2006) 450:76–85. 10.1111/j.1651-2227.2006.tb02378.x 16817681

[32] Tanner JM ed. Growth at adolescence, Second Edition. Oxford, UK: Blackwell Scientific (1962).

[33] Danne T Nimri R Battelino T Bergenstal RM Close KL DeVries JH International Consensus on use of continuous glucose monitoring. Diabetes Care (2017) 40:1631–40. 10.2337/dc17-1600 PMC6467165 29162583

[34] Battelino T Danne T Bergenstal RM Amiel SA Beck R Biester T Clinical targets for continuous glucose monitoring data interpretation: recommendations from the International Consensus on time in range. Diabetes Care (2019) 42:1593–603. 10.2337/dci19-0028 PMC6973648 31177185

[35] Clarke W Jones T Rewers A Dunger D Klingensmith GJ Assessment and management of hypoglycemia in children and adolescents with diabetes. Pediatr Diabetes (2008) 9:165–74. 10.1111/j.1399-5448.2008.00405.x 18416698

[36] Dunger DB Sperling MA Acerini CL Bohn DJ Daneman D Danne TP ESPE/LWPES consensus statement on diabetic ketoacidosis in children and adolescents. Arch Dis Child (2004) 89:188–94. 10.1136/adc.2003.044875 PMC1719805 14736641

[37] Chen P Mao L Nassis GP Harmer P Ainsworth BE Li F Coronavirus disease (COVID-19): the need to maintain regular physical activity while taking precautions. J Sport Health Sci (2020) 9:103–4. 10.1016/j.jshs.2020.02.001 PMC7031771 32099716

[38] Verma A Rajput R Verma S Balania VKB Jangra B Impact of lockdown in COVID 19 on glycemic control in patients with type 1 diabetes mellitus. Diabetes Metab Syndr (2020) 14:1213–6. 10.1016/j.dsx.2020.07.016 PMC7357511 32679527

[39] American Diabetes Association. 13 Children and adolescents: standards of medical care in diabetes-2020. Diabetes Care (2020) 43:S163–82. 10.2337/dc20-S013 31862756

[40] Diabetes Control and Complications Trial Research Group Effect of intensive diabetes treatment on the development and progression of long-term complications in adolescents with insulin-dependent diabetes mellitus: Diabetes Control and Complications Trial. J Pediatr (1994) 125:177–88. 10.1016/s0022-3476(94)70190-3 8040759

[41] Capaldo B Annuzzi G Creanza A Giglio C De Angelis R Lupoli R Blood glucose control during lockdown for COVID-19: CGM metrics in Italian adults with type 1 diabetes. Diabetes Care (2020) 43:e88–9. 10.2337/dc20-1127 PMC7372051 32540921

[42] Wu J Huang J Zhu G Wang Q Lv Q Huang Y Elevation of blood glucose level predicts worse outcomes in hospitalized patients with COVID-19: a retrospective cohort study. BMJ Open Diabetes Res Care (2020) 8:e001476. 10.1136/bmjdrc-2020-001476 PMC7298690 32503812

[43] Ebekozien OA Noor N Gallagher MP Alonso GT Type 1 diabetes and COVID-19: preliminary findings from a multicenter surveillance study in the U.S. Diabetes Care (2020) 43:e83–5. 10.2337/dc20-1088 PMC7372041 32503837

[44] Fadini GP Morieri ML Longato E Avogaro A Prevalence and impact of diabetes among people infected with SARS-CoV-2. J Endocrinol Invest (2020) 43:867–9. 10.1007/s40618-020-01236-2 PMC7103097 32222956

[45] Guo W Li M Dong Y Zhou H Zhang Z Tian C Diabetes is a risk factor for the progression and prognosis of COVID-19. Diabetes Metab Res Rev (2020) e3319. 10.1002/dmrr.3319 PMC7228407 32233013

[46] Jackson CC Albanese-O’Neill A Butler KL Chiang JL Deeb LC Hathaway K Diabetes care in the school setting: a position statement of the American Diabetes Association. Diabetes Care (2015) 38:1958–63. 10.2337/dc15-1418 26404925

[47] Bratina N Forsander G Annan F Wysocki T Pierce J Calliari LE ISPAD Clinical Practice Consensus Guidelines 2018: Management and support of children and adolescents with type 1 diabetes in school. Pediatr Diabetes (2018) 19:287–301. 10.1111/pedi.12743 30084519

[48] Burckhardt MA Fried L Bebbington K Hancock M Nicholas JA Roberts A Use of remote monitoring with continuous glucose monitoring in young children with type 1 diabetes: the parents’ perspective. Diabetes Med (2019) 36:1453–9. 10.1111/dme.14061 31257642

[49] Garvey K Wolfsdorf JI The impact of technology on current diabetes management. Pediatr Clin North Am (2015) 62:873–88. 10.1016/j.pcl.2015.04.005 26210622

[50] Raymond J Updates in behavioural and psychosocial literature in adolescents with type 1 diabetes. Curr Opin Endocrinol Diabetes Obes (2015) 22:265–9. 10.1097/MED.0000000000000167 26087340

[51] Foster NC Beck RW Miller KM Clements MA Rickels MR DiMeglio LA State of type 1 diabetes management and outcomes from the T1D Exchange in 2016-2018. Diabetes Technol Ther (2019) 21:66–72. 10.1089/dia.2018.0384 30657336 PMC7061293

[52] Burckhardt MA Smith GJ Cooper MN Jones TW Davis EA Real-world outcomes of insulin pump compared to injection therapy in a population-based sample of children with type 1 diabetes. Pediatr Diabetes (2018) 19:1459–66. 10.1111/pedi.12754 30129154

[53] Christoforidis A Kavoura E Nemtsa A Pappa K Dimitriadou M Coronavirus lockdown effect on type 1 diabetes management on children wearing insulin pump equipped with continuous glucose monitoring system. Diabetes Res Clin Pract (2020) 166:108307. 10.1016/j.diabres.2020.108307 32650036 PMC7340587

[54] Lee SWH Ooi L Lai YK Telemedicine for the management of glycemic control and clinical outcomes of type 1 diabetes mellitus: a systematic review and meta-analysis of randomized controlled studies. Front Pharmacol (2017) 8:330:330. 10.3389/fphar.2017.00330 28611672 PMC5447671

[55] Griffith ML Siminerio L Payne T Krall J A shared decision-making approach to telemedicine: engaging rural patients in glycemic management. J Clin Med (2016) 5:103. 10.3390/jcm5110103 PMC5126800 27869655

[56] Sardu C Santamaria M Rizzo MR Barbieri M di Marino M Paolisso G Telemonitoring in heart failure patients treated by cardiac resynchronisation therapy with defibrillator (CRT-D): the TELECART Study. Int J Clin Pract (2016) 70:569–76. 10.1111/ijcp.12823 PMC5813682 27291327

[57] Savarino EV Iovino P Santonicola A Ghisa M Laserra G Barberio B Clinical and psychological impact of COVID-19 infection in adult patients with eosinophilic gastrointestinal disorders during the SARS-CoV-2 outbreak. J Clin Med (2020) 9:2011. 10.3390/jcm9062011 PMC7355569 32604895

[58] Young JD Badowski ME Telehealth: increasing access to high quality care by expanding the role of technology in correctional medicine. J Clin Med (2017) 6:20. 10.3390/jcm6020020 PMC5332924 28208807

[59] Terheggen U Heiring C Kjellberg M Hegardt F Kneyber M Gente M European consensus recommendations for neonatal and paediatric retrievals of positive or suspected COVID-19 patients. Pediatr Res (2020). 10.1038/s41390-020-1050-z 32634819

[60] Castagnoli R Votto M Licari A Brambilla I Bruno R Perlini S Severe Acute Respiratory Syndrome Coronavirus 2 (SARS-CoV-2) infection in children and adolescents: a systematic review. JAMA Pediatr (2020) 174:882–9. 10.1001/jamapediatrics.2020.1467 32320004

[61] Dong Y Mo X Hu Y Qi X Jiang F Jiang Z Epidemiology of COVID-19 among children in China. Pediatrics (2020) 145:e20200702. 10.1542/peds.2020-0702 32179660

[62] Götzinger F Santiago-García B Noguera-Julián A Lanaspa M Lancella L Calò Carducci FI COVID-19 in children and adolescents in Europe: a multinational, multicentre cohort study. Lancet Child Adolesc Health (2020) 4:653–61. 10.1016/S2352-4642(20)30177-2 PMC7316447 32593339

[63] Yasuhara J Kuno T Takagi H Sumitomo N Clinical characteristics of COVID-19 in children: a systematic review. Pediatr Pulmonol (2020). 10.1002/ppul.24991 32725955

[64] International Society of Pediatric and Adolescent Diabetes (ISPAD) Summary of recommendations regarding COVID-19 in children with diabetes. Available at: https://www.ispad.org/page/CoronavirusinfectionCOVID-19 (Accessed August 3, 2020).

[65] International Society of Pediatric and Adolescent Diabetes (ISPAD) Summary of recommendations regarding COVID-19 in children with diabetes: keep calm and mind your diabetes care and public health advice. Pediatr Diabetes (2020) 21:413–4. 10.1111/pedi.13013 PMC7267149 32346988

[66] Grimsmann JM von Sengbusch S Freff M Ermer U Placzek K Danne T Glucose management indicator based on sensor data and laboratory HbA1c in people with type 1 diabetes from the DPV database: differences by sensor type. Diabetes Care (2020) 43:e111–2. 10.2337/dc20-0259 32690487

[67] Calhoun P Johnson TK Hughes J Price D Balo AK Resistance to acetaminophen interference in a novel continuous glucose monitoring system. J Diabetes Sci Technol (2018) 12:393–6. 10.1177/1932296818755797 PMC5851234 29334775

[68] Monaghan M Marks B Personal experiences with COVID-19 and diabetes technology: all for technology yet not technology for all. J Diabetes Sci Technol (2020) 14:762–3. 10.1177/1932296820930005 PMC7673164 32460543

