# Supplementary material for: Glycemic Control Improvement in Italian Children and Adolescents With Type 1 Diabetes Followed Through Telemedicine During Lockdown Due to the COVID-19 Pandemic
Source: Front Endocrinol (Lausanne). 2020 Dec 7;11:595735. doi: 10.3389/fendo.2020.595735 (PMC7793913; doi:10.3389/fendo.2020.595735)
Supplement: Supplementary file 1 [file Table_1.docx]

**Table S1. Clinic, therapy, and glycemic control parameters in children and adolescents with T1D according to gender**

| **Variable** | **Males (n = 31)** | | | **Females (n = 31)** | | |
| --- | --- | --- | --- | --- | --- | --- |
|  | **T0** | **T1** | **p** | **T0** | **T1** | **p** |
| **Age (years)** | 11.1 ± 4.74 (11.0) | 11.4 ± 4.74 (11.3) | **<0.0001** | 11.0 ± 4.05 (10.3) | 11.3 ± 4.05 (10.6) | **<0.0001** |
| **T1D time disease (years)** | 5.37 ± 4.49 (4.42) | 5.67 ± 4.49 (4.72) | **<0.0001** | 4.42 ± 3.96 (2.75) | 4.72 ± 3.96 (3.05) | **<0.0001** |
| **Height (SDS)** | -0.23 ± 1.05 (-0.27) | -- | -- | 0.34 ± 0.90 (0.36) ***** | -- | -- |
| **BMI z-score (SDS)** | -0.08 ± 0.92 (-0.04) | -- | -- | 0.00 ± 1.08 (0.13) | -- | -- |
| **Puberty (NO/YES) (%)** | 18/13 (58.1/41.9) | 18/13 (58.1/41.9) | -- | 16/15 (51.6/48.4) | 16/15 (51.6/48.4) | χ^2^ = 0.26; p = 0.610 |
| **Insulin delivery method (MDI/CSII) (%)** | 15/16 (48.4/51.6) | 15/16 (48.4/51.6) | -- | 18/13 (58.1/41.9) | 18/13 (58.1/41.9) | χ^2^ = 0.58; p = 0.445 |
| **Meal-Time insulin (IU/kg/day)** | 0.36 ± 0.14 (0.35) | 0.39 ± 0.14 (0.37) | **0.006** | 0.44 ± 0.13 (0.43) ***** | 0.41 ± 0.12 (0.39) | 0.198 |
| **Basal insulin (IU/kg/day)** | 0.30 ± 0.11 (0.33) | 0.33 ± 0.11 (0.32) | **0.022** | 0.34 ± 0.13 (0.33) | 0.36 ± 0.14 (0.35) | 0.316 |
| **TDD insulin (IU/kg/day)** | 0.66 ± 0.22 (0.68) | 0.71 ± 0.21 (0.72) | **0.002** | 0.78 ± 0.21 (0.81) ***** | 0.76 ± 0.18 (0.78) | 0.375 |
| **Physical Activity (h/week)** | 4.00 ± 3.25 (4.00) | 0.29 ± 0.53 (0.00) | **<0.0001** | 2.53 ± 2.12 (2.00) | 0.19 ± 0.65 (0.00) | **<0.0001** |
| **GMI (%)**  **GMI (mmol/mol)** | 7.44 ± 0.73 (7.30)  57.8 ± 7.97 (56.3) | 7.35 ± 0.75 (7.20)  56.8 ± 8.22 (55.2) | 0.172  0.165 | 7.46 ± 0.77 (7.50)  58.0 ± 8.42 (58.5) | 7.36 ± 0.70 (7.50)  56.9 ± 7.69 (58.5) | 0.246  0.269 |
| **GMI ≤7.0% (YES/NO) (%)** | 11/20 (35.5/64.5) | 12/19 (38.7/61.3) | χ^2^ = 0.07; p = 0.791 | 11/20 (35.5/64.5) | 9/22 (29.0/71.0) | χ^2^ = 0.07; p = 0.786 |
| **Average glucose (mg/dl)** | 167.3 ± 21.6 (162.0) | 164.8 ± 22.2 (160.0) | 0.160 | 167.5 ± 22.1 (169.0) | 164.4 ± 20.3 (168.0) | 0.213 |
| **SD glucose (mg/dl)** | 62.3 ± 13.4 (65.0) | 58.8 ± 12.6 (62.0) | **<0.001** | 59.4 ± 10.0 (61.0) | 56.4 ± 8.64 (58.0) | **<0.001** |
| **%CV (%)** | 37.0 ± 5.31 (36.1) | 35.4 ± 4.85 (35.2) | **0.007** | 35.6 ± 5.30 (36.2) | 34.3 ± 4.98 (34.0) | **0.033** |
| **TBR^<70^ (%)**  **TBR^<54^ (%)** | 2.68 ± 2.34 (1.90)  0.56 ± 0.77 (0.30) | 2.11 ± 1.85 (1.50)  0.36 ± 0.52 (0.20) | **0.038**  0.216 | 2.58 ± 2.44 (1.80)  0.45 ± 0.44 (0.30) | 2.15 ± 2.89 (1.30)  0.32 ± 0.55 (0.20) | **0.012**  **0.004** |
| **TIR^70-180^ (%)** | 60.9 ± 13.3 (62.3) | 62.9 ± 15.0 (65.4) | **0.047** | 59.2 ± 13.1 (56.7) | 61.4 ± 12.5 (63.3) | 0.083 |
| **TAR^>180^ (%)**  **TAR^>250^ (%)** | 37.3 ± 13.5 (34.5)  11.7 ± 8.87 (9.70) | 35.0 ± 15.3 (32.4)  10.1 ± 8.22 (7.60) | 0.136  **0.018** | 38.2 ± 14.4 (41.2)  11.2 ± 6.71 (10.3) | 36.4 ± 13.6 (36.1)  9.40 ± 5.75 (9.70) | 0.193  **0.006** |
| **Sensor usage (%)** | 92.1 ± 10.7 (96.2) | 90.9 ± 13.8 (96.2) | 0.813 | 92.0 ± 12.1 (97.3) | 92.7 ± 10.2 (97.0) | 0.499 |

Continuous data are reported as mean ± SD (median); categorical as absolute frequencies (percent values). Abbreviations: BMI, body mass index; CSII, continuous subcutaneous insulin infusion; CV, coefficient of variation; GMI, glucose management indicator; MDI, multiple daily injections; SD, standard deviation; SDS, standard deviation score; T1D, type 1 diabetes; TAR, time above range; TBR, time below range; TDD, total daily dose; TIR, time in range. *p<0.01 Mann-Whitney U Test between groups at the same period time

**Table S2. Clinic, therapy, and glycemic control parameters in children and adolescents with T1D according to pubertal status**

| **Variable** | **Puberty NO (n = 34)** | | | **Puberty YES (n = 28)** | | |
| --- | --- | --- | --- | --- | --- | --- |
|  | **Period T0** | **Period T1** | **p** | **Period T0** | **Period T1** | **p** |
| **Gender (Males/Females) (%)** | 18/16 (52.9/47.1) | 18/16 (52.9/47.1) | -- | 13/15 (46.4/53.6) | 13/15 (46.4/53.6) | χ^2^ = 0.26; p = 0.610 |
| **Age (years)** | 7.94 ± 2.71 (8.85) | 8.24 ± 2.71 (9.15) | **<0.0001** | 14.9 ± 2.57 (14.6) **^** | 15.2 ± 2.56 (14.9) **^** | **<0.0001** |
| **T1D time disease (years)** | 2.74 ± 2.18 (2.25) | 3.04 ± 2.18 (2.55) | **<0.0001** | 7.51 ± 4.65 (7.42) **^** | 7.80 ± 4.65 (7.72) **^** | **<0.0001** |
| **Height (SDS)** | -0.12 ± 0.99 (0.15) | -- | -- | 0.26 ± 1.02 (0.12) | -- | 0.218 |
| **BMI z-score (SDS)** | -0.14 ± 0.98 (-0.25) | -- | -- | 0.09 ± 1.02 (0.32) | -- | 0.246 |
| **Insulin delivery method (MDI/CSII)** | 23/11 (67.6/32.4) | 23/11 (67.6/32.4) | -- | 10/18 (35.7/64.3) | 10/18 (35.7/64.3) | χ^2^ = 6.29; **p = 0.012** |
| **Meal-Time insulin (IU/kg/day)** | 0.39 ± 0.15 (0.37) | 0.37 ± 0.13 (0.37) | 0.776 | 0.42 ± 0.13 (0.40) | 0.43 ± 0.13 (0.39) | 0.517 |
| **Basal insulin (IU/kg/day)** | 0.28 ± 0.12 (0.30) | 0.29 ± 0.13 (0.28) | 0.517 | 0.37 ± 0.11 (0.37) ***** | 0.39 ± 0.11 (0.38) ***** | **0.003** |
| **TDD insulin (IU/kg/day)** | 0.67 ± 0.23 (0.68) | 0.67 ± 0.18 (0.70) | 0.551 | 0.79 ± 0.19 (0.78) **#** | 0.82 ± 0.18 (0.83) ***** | 0.167 |
| **Physical Activity (h/week)** | 2.44 ± 2.19 (2.00) | 0.21 ± 0.59 (0.00) | **<0.0001** | 4.27 ± 3.19 (4.00) **#** | 0.29 ± 0.60 (0.00) | **<0.0001** |
| **GMI (%)**  **GMI (mmol/mol)** | 7.27 ± 0.72 (7.30)  55.9 ± 7.90 (56.3) | 7.13 ± 0.66 (7.20)  54.4 ± 7.21 (55.2) | 0.052  0.063 | 7.66 ± 0.72 (7.85)  60.3 ± 7.89 (62.3) | 7.63 ± 0.71 (7.75) **#**  59.9 ± 7.76 (61.2) **#** | 0.617  0.558 |
| **GMI ≤7.0% (YES/NO) (%)** | 15/19 (44.1/55.9) | 14/20 (41.2/58.8) | χ^2^ = 0.06; p = 0.806 | 7/21 (25.0/75.0) | 7/21 (25.0/75.0) | χ^2^ = 0.00; p = 1.000 |
| **Average glucose (mg/dl)** | 162.2 ± 20.9 (162.0) | 158.0 ± 19.2 (159.5) | **0.033** | 173.6 ± 21.2 (178.5) | 172.7 ± 20.8 (175.5) **#** | 0.589 |
| **SD glucose (mg/dl)** | 60.7 ± 11.8 (59.5) | 57.5 ± 10.8 (56.0) | **<0.001** | 61.0 ± 12.0 (63.0) | 57.7 ± 11.0 (59.0) | **<0.001** |
| **%CV (%)** | 37.4 ± 4.99 (37.1) | 36.2 ± 5.07 (35.1) | **0.042** | 35.1 ± 5.49 (35.0) | 33.3 ± 5.49 (33.2) **#** | **0.004** |
| **TBR^<70^ (%)**  **TBR^<54^ (%)** | 3.16 ± 2.42 (2.70)  0.59 ± 0.65 (0.35) | 2.87 ± 2.91 (2.00)  0.49 ± 0.67 (0.30) | 0.073  0.142 | 1.99 ± 2.18 (1.30) **#**  0.40 ± 0.6 (0.15) **#** | 1.24 ± 1.12 (1.10) *****  0.17 ± 0.20 (0.10) ***** | **0.003**  **0.003** |
| **TIR^70-180^ (%)** | 62.7 ± 12.3 (61.9) | 65.6 ± 11.4 (65.7) | **0.003** | 56.8 ± 13.6 (53.6) | 57.9 ± 15.3 (53.9) **#** | 0.393 |
| **TAR^>180^ (%)**  **TAR^>250^ (%)** | 34.1 ± 12.9 (34.3)  10.6 ± 7.62 (9.00) | 31.5 ± 12.1 (32.0)  8.41 ± 6.36 (7.60) | **0.024**  **<0.001** | 42.3 ± 13.9 (47.3) **#**  12.5 ± 8.00 (10.5) | 40.8 ± 15.5 (43.7) **#**  11.4 ± 7.51 (10.5) | 0.682  0.143 |
| **Sensor usage (%)** | 93.9 ± 10.1 (97.2) | 94.2 ± 10.7 (97.0) | 0.401 | 89.8 ± 12.5 (94.6) | 88.9 ± 13.2 (94.3) **#** | 0.716 |

Continuous data are reported as mean ± SD (median); categorical as absolute frequencies (percent values). Abbreviations: BMI, body mass index; CSII, continuous subcutaneous insulin infusion; CV, coefficient of variation; GMI, glucose management indicator; MDI, multiple daily injections; SD, standard deviation; SDS, standard deviation score; T1D, type 1 diabetes; TAR, time above range; TBR, time below range; TDD, total daily dose; TIR, time in range. #p<0.05, *p<0.01, ^p<0.0001 Mann-Whitney U Test between groups at the same period time

**Table S3. Clinic, therapy, and glycemic control parameters in children and adolescents with T1D according to insulin delivery method**

| **Variable** | **MDI (n = 33)** | | | | **CSII (n = 29)** | | |
| --- | --- | --- | --- | --- | --- | --- | --- |
|  | **Period T0** | **Period T1** | **p** | **Period T0** | | **Period T1** | **p** |
| **Gender (Males/Females) (%)** | 15/18 (45.5/54.5) | 15/18 (45.5/54.5) | -- | 16/13 (55.2/44.8) | | 16/13 (55.2/44.8) | χ^2^ = 0.58; p = 0.445 |
| **Age (years)** | 9.41 ± 4.24 (9.44) | 9.71 ± 4.24 (9.73) | **<0.0001** | 13.0 ± 3.74 (13.0) **^** | | 13.3 ± 3.74 (13.3) **^** | **<0.0001** |
| **T1D time disease (years)** | 2.77 ± 3.21 (1.25) | 3.07 ± 3.21 (1.55) | **<0.0001** | 7.31 ± 3.97 (5.75) **^** | | 7.61 ± 3.97 (6.05) **^** | **<0.0001** |
| **Height (SDS)** | -0.03 ± 0.98 (0.00) | -- | -- | 0.15 ± 1.05 (0.16) | | -- | 0.549 |
| **BMI z-score (SDS)** | -0.16 ± 1.11 (-0.09) | -- | -- | 0.11 ± 0.83 (0.20) | | -- | 0.413 |
| **Puberty (NO/YES) (%)** | 23/10 (69.7/30.3) | 23/10 (69.7/30.3) | -- | 11/18 (37.9/62.1) | | 11/18 (37.9/62.1) | χ^2^ = 6.29; **p = 0.012** |
| **Meal-Time insulin (IU/kg/day)** | 0.40 ± 0.15 (0.41) | 0.41 ± 0.13 (0.39) | 0.267 | 0.40 ± 0.13 (0.37) | | 0.38 ± 0.13 (0.36) | 0.665 |
| **Basal insulin (IU/kg/day)** | 0.26 ± 0.12 (0.27) | 0.27 ± 0.11 (0.25) | 0.329 | 0.39 ± 0.10 (0.39) **^** | | 0.42 ± 0.11 (0.44) **^** | **0.023** |
| **TDD insulin (IU/kg/day)** | 0.66 ± 0.23 (0.69) | 0.68 ± 0.20 (0.71) | 0.262 | 0.79 ± 0.18 (0.78) **^** | | 0.80 ± 0.17 (0.80) | 0.509 |
| **Physical Activity (h/week)** | 2.88 ± 3.06 (2.00) | 0.30 ± 0.68 (0.00) | **<0.0001** | 3.71 ± 2.49 (4.00) | | 0.17 ± 0.47 (0.00) | **<0.0001** |
| **GMI (%)**  **GMI (mmol/mol)** | 7.44 ± 0.79 (7.50)  57.8 ± 8.58 (58.5) | 7.31 ± 0.71 (7.40)  56.3 ± 7.73 (57.4) | 0.071  0.081 | 7.45 ± 0.71 (7.30)  57.9 ± 7.73 (56.3) | | 7.41 ± 0.75 (7.20)  57.4 ± 8.17 (55.2) | 0.518  0.476 |
| **GMI ≤7.0% (YES/NO) (%)** | 11/22 (33.3/66.7) | 11/22 (33.3/66.7) | χ^2^ = 0.00; p = 1.000 | 11/18 (37.9/62.1) | | 10/19 (34.5/65.5) | χ^2^ = 0.07; p = 0.785 |
| **Average glucose (mg/dl)** | 167.1 ± 22.8 (169.0) | 163.2 ± 20.7 (166.0) | 0.079 | 167.7 ± 20.7 (162.0) | | 166.3 ± 21.9 (161.0) | 0.374 |
| **SD glucose (mg/dl)** | 59.2 ± 12.7 (57.0) | 56.7 ± 11.8 (55.0) | **0.005** | 62.7 ± 10.6 (64.0) | | 58.6 ± 9.61 (59.0) | **<0.001** |
| **%CV (%)** | 35.4 ± 5.55 (35.4) | 34.7 ± 5.97 (33.5) | 0.308 | 37.4 ± 4.88 (38.0) | | 35.0 ± 3.40 (34.3) | **<0.001** |
| **TBR^<70^ (%)**  **TBR^<54^ (%)** | 2.20 ± 2.19 (1.60)  0.32 ± 0.38 (0.20) | 2.26 ± 3.02 (1.40)  0.38 ± 0.70 (0.20) | 0.557  0.819 | 3.11 ± 2.5 (1.90)  0.71 ± 0.78 (0.40) **#** | | 1.98 ± 1.48 (1.50)  0.30 ± 0.24 (0.20) | **<0.0001**  **<0.001** |
| **TIR^70-180^ (%)** | 60.9 ± 14.3 (61.0) | 63.0 ± 13.1 (62.9) | 0.080 | 59.0 ± 11.8 (60.5) | | 61.2 ± 14.6 (63.8) | 0.058 |
| **TAR^>180^ (%)**  **TAR^>250^ (%)** | 36.8 ± 14.9 (37.4)  11.5 ± 8.36 (9.85) | 34.7 ± 13.8 (36.1)  9.31 ± 6.87 (9.35) | 0.114  **<0.001** | 38.9 ± 12.8 (37.2)  11.3 ± 7.17 (10.1) | | 36.8 ± 15.2 (32.9)  10.2 ± 7.25 (8.10) | 0.234  0.103 |
| **Sensor usage (%)** | 94.9 ± 7.99 (97.3) | 94.4 ± 9.27 (96.8) | 0.181 | 88.8 ± 13.7 (94.6) **#** | | 88.9 ± 14.3 (95.5) | 0.084 |

Continuous data are reported as mean ± SD (median); categorical as absolute frequencies (percent values). Abbreviations: BMI, body mass index; CSII, continuous subcutaneous insulin infusion; CV, coefficient of variation; GMI, glucose management indicator; MDI, multiple daily injections; SD, standard deviation; SDS, standard deviation score; T1D, type 1 diabetes; TAR, time above range; TBR, time below range; TDD, total daily dose; TIR, time in range. #p<0.05, ^p<0.0001 Mann-Whitney U Test between groups at the same period time
